# SARS-CoV-2 Mpro inhibitors from *Siphonostegia chinensis*: an integrated biophysical and computational study

**DOI:** 10.3389/fchem.2026.1790228

**Published:** 2026-04-10

**Authors:** Yingjie Ren, Wei Wang, Dai Zhang, Mingliang Zhang, Hui Zhang, Yali Wu, Feiyan Liu, Liuqing Yang, Pan Wang, Lei Chen, Xiaofei Chen, Jinfa Tang, Xianqing Ren

**Affiliations:** 1 Department of Pharmacy, the First Affiliated Hospital of Henan University of Chinese Medicine, Zhengzhou, Henan, China; 2 Henan Province Engineering Research Center for Clinical Application, Evaluation and Transformation of Traditional Chinese Medicine, Henan Provincial Key Laboratory for Clinical Pharmacy of Traditional Chinese Medicine, Henan Province Engineering Research Center of Safety Evaluation and Risk Management of Traditional Chinese Medicine, Zhengzhou, Henan, China; 3 Department of Disease Prevention and Control, The First Affiliated Hospital of Henan University of Chinese Medicine, Zhengzhou, Henan, China; 4 School of Pharmacy, Henan University of Chinese Medicine, Zhengzhou, Henan, China; 5 Collaborative Innovation Center of Prevention and Treatment of Major Diseases by Chinese and Western Medicine, Zhengzhou, Henan, China; 6 Hospital of Pediatrics, The First Affiliated Hospital of Henan University of Traditional Chinese Medicine, Zhengzhou, Henan, China; 7 Pediatric Medical College, Henan University of Traditional Chinese Medicine, Zhengzhou, Henan, China

**Keywords:** molecular dynamics, Mpro inhibitors, SARS-CoV-2, *Siphonostegia chinensis* Benth, UPLC-Q- TOF-MS

## Abstract

**Background:**

The COVID-19 pandemic caused by SARS-CoV-2 underscores the urgent need for novel antiviral agents. Preliminary screening indicated that the traditional Chinese medicine *Siphonostegia chinensis* Benth. (Bei Liu Ji Nu) possesses inhibitory activity against the SARS-CoV-2 main protease (Mpro), a key enzyme for viral replication.

**Methods:**

To identify the active constituents responsible for its anti-SARS-CoV-2 activity, we employed an integrated strategy combining bio-layer interferometry (BLI) and ultra-performance liquid chromatography coupled with quadrupole time-of-flight mass spectrometry (UPLC-QTOF-MS).

**Results:**

Two phenolic compounds, verbascoside and 3,4-dicaffeoylquinic acid, were successfully fished out and identified from the ethanol extract as the most potent binders to Mpro. The binding affinities (KD) were determined to be 2.149 × 10^−6^ M and 2.487 × 10^−5^ M, respectively. Subsequent *in vitro* enzymatic inhibition assays confirmed their inhibitory effects, with IC_50_ values of 0.076 µM and 0.194 µM, respectively. To elucidate the inhibition mechanism, molecular docking and molecular dynamics simulations were performed, revealing stable binding modes of both compounds within the catalytic pocket of Mpro through key interactions. Preliminary ADMET (Absorption, Distribution, Metabolism, Excretion, and Toxicity) predictions were also conducted to assess their drug-likeness.

**Conclusion:**

Our findings demonstrate that verbascoside and 3,4-dicaffeoylquinic acid exhibit high binding affinity and structural stability against SARS-CoV-2 Mpro, highlighting their potential as lead compounds for inhibiting viral replication. Furthermore, the comprehensive strategy established in this study provides a reliable and efficient approach for identifying bioactive components from complex herbal matrices, offering new perspectives and a scientific basis for the development of natural product-derived targeted therapeutics.

## 1 Introduction

The global pandemic of coronavirus disease 2019 (COVID-19), caused by severe acute respiratory syndrome coronavirus 2 (SARS-CoV-2) (Mohammadi et al., 2024), continues to evolve worldwide with the persistent emergence of viral variants, highlighting the urgency for controlling emerging infectious diseases (Anton et al., 2024; Balapasheva et al., 2025; Zhang Y. et al., 2025). This pandemic has also spurred increased scientific interest in exploring antiviral agents from natural sources (Duan et al., 2023). Compared to modern synthetic drugs, systematic research on the anti-coronavirus activities of natural compounds remains relatively limited in the early stages (Banerjee et al., 2021). The 3-chymotrypsin-like protease (3CLpro, also known as the main protease, Mpro) encoded by the SARS-CoV-2 genome is a pivotal enzyme in viral replication (Zagórska et al., 2024; Lv et al., 2022). Belonging to the cysteine protease family, its highly conserved catalytic site is recognized as a crucial target for developing broad-spectrum anti-coronavirus drugs (Wang et al., 2025).

Against this backdrop, discovering potent and safe anti-SARS-CoV-2 active ingredients from traditional medicinal plants has become a pressing and promising research direction (Silva et al., 2021). *Siphonostegia chinensis* Benth. (Bei Liu Ji Nu), the dried entire herb of *Siphonostegia chinensis* Benth. (family Scrophulariaceae, also known as Jin Zhong Yin Chen or Huang Hua Yin Chen), is a commonly used traditional Chinese medicine (Huang et al., 2024). Modern pharmacological studies have revealed that its extracts are rich in flavonoids, phenylethanoid glycosides, and lactones, exhibiting significant antibacterial and anti-inflammatory activities, particularly in alleviating inflammation-related pathological states (Zhang et al., 2022). This suggests its potential to contain active components that may intervene in viral infections. However, whether this herb possesses the potential to inhibit key SARS-CoV-2 proteins awaits in-depth exploration.

**FIGURE 1:**
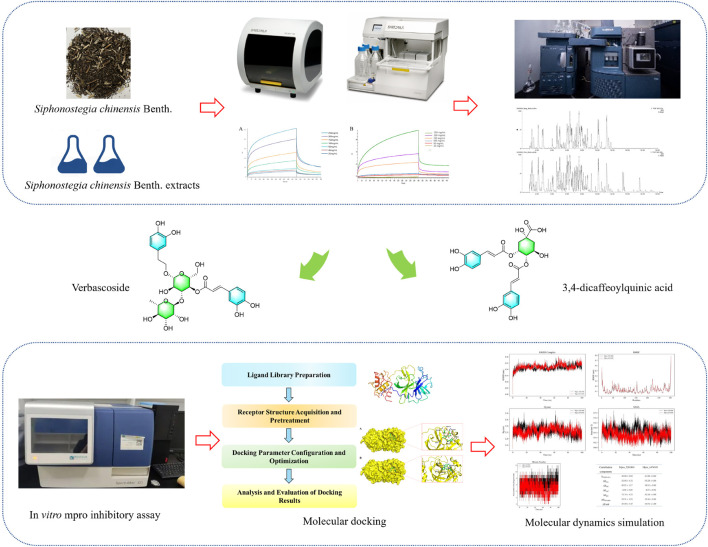
The workflow of this study. The integrated strategy for identifying Mpro inhibitors from Siphonostegia chinensis Benth. includes extraction, bioactivity screening, ligand fishing by BLI, compound identification by UPLC-Q-TOF-MS, *in vitro* validation, and computational simulations.

Rapidly identifying active ingredients that interact with specific targets within complex traditional Chinese medicine systems remains a significant technical challenge. Conventional isolation methods are often cumbersome, inefficient, and lack specificity. In this study, we established an efficient screening system by coupling Bio-Layer Interferometry (BLI) with ultra-performance liquid chromatography-quadrupole-time-of-flight mass spectrometry (UPLC-Q-TOF-MS). This system enables the highly sensitive and specific fishing and identification of potential Mpro inhibitors from herbal extracts. The activity of fished compounds was further validated by *in vitro* enzymatic inhibition assays. Their mechanisms of action were investigated using molecular docking and molecular dynamics simulations (Shi et al., 2023). This integrated approach allows for the systematic and rapid discovery of antiviral components targeting SARS-CoV-2 Mpro from Siphonostegia chinensis Benth., providing both candidate compounds and methodological references for anti-COVID-19 drug development. The overall research workflow is illustrated in Figure 1.

## 2 Materials and methods

### 2.1 Plant materials and preparation of extracts

The dried herb of Siphonostegia chinensis Benth. (Bei Liu Ji Nu) was provided by Sichuan New Green Pharmaceutical Technology Development Co., Ltd. (China). The powdered herb was subjected to extraction using two methods: For the ethanol extract, the powder was refluxed with 70% ethanol (liquid-to-solid ratio of 60 mL/g) at 80 °C for 2 h. This extraction process was repeated twice. The combined filtrates were concentrated under reduced pressure and subsequently lyophilized or spray-dried to obtain the ethanol extract powder. For the aqueous extract, the powdered herb was decocted with water to prepare a decoction for subsequent use.

### 2.2 Expression and purification of recombinant SARS-CoV-2 Mpro

#### 2.2.1 Plasmid construction and strain

The full-length gene sequence of SARS-CoV-2 Mpro (GenBank: NC_045512) was codon-optimized for *Escherichia coli* K12 and synthesized by GenScript Biotech Co., Ltd. (Jiangsu, China). A sequence encoding an N-terminal 6×His-tag was incorporated into the 5′-end of the optimized gene. The His-tagged Mpro gene was then cloned into the pET22b expression vector and transformed into competent *E. coli* BL21 (DE3) Gold cells for protein expression.

#### 2.2.2 Protein expression and purification

The expression and purification of recombinant SARS-CoV-2 Mpro were performed with modifications based on a previously described method (Zhang D. et al., 2025). Briefly, a single colony harboring the recombinant plasmid was inoculated into LB medium containing ampicillin (100 μg/mL) and grown overnight at 37 °C with shaking to prepare the seed culture. The seed culture was then diluted 1:100 into 4 L of fresh LB medium with ampicillin and grown at 37 °C until the optical density at 600 nm (OD600) reached 0.6–0.8. Protein expression was induced by adding isopropyl β-D-1-thiogalactopyranoside (IPTG) to a final concentration of 0.2 mM, and the culture was further incubated at 18 °C for 16 h with shaking. Cells were harvested by centrifugation at 4 °C. The cell pellet was resuspended in lysis buffer (50 mM Tris-HCl, 300 mM NaCl, 10 mM imidazole, 5% glycerol, 1 mM TCEP, pH 7.5) supplemented with a commercial EDTA-free protease inhibitor cocktail. Cell lysis was performed using a high-pressure homogenizer on an ice-water bath. The lysate was centrifuged at 12,000 × g for 40 min at 4 °C, and the supernatant was filtered through a 0.45 μm membrane to obtain a clarified lysate. The clarified lysate was loaded onto a Ni-NTA affinity resin column pre-equilibrated with the lysis buffer. The column was sequentially washed with wash buffer I (50 mM Tris-HCl, 300 mM NaCl, 20 mM imidazole, 5% glycerol, 1 mM TCEP, pH 7.5) and wash buffer II (containing 50 mM imidazole). The bound His-tagged Mpro protein was eluted with an elution buffer containing 300 mM imidazole. The fractions containing Mpro were pooled and concentrated (Supplementary Figure S1). Further purification was achieved by size-exclusion chromatography (SEC) on a Superdex 75 Increase 10/300 GL column pre-equilibrated with storage buffer (20 mM Tris-HCl, 100 mM NaCl, 1 mM TCEP, pH 7.5). The fractions corresponding to the monomeric Mpro peak were collected. The purified protein was concentrated to 10 mg/mL, aliquoted, and stored at −80 °C for subsequent experiments.

### 2.3 Validation of Mpro inhibitory activity using a FRET-based assay

To systematically evaluate the inhibitory potential of *Siphonostegia chinensis* Benth. extracts and their active constituents against SARS-CoV-2 Mpro, a fluorescence resonance energy transfer (FRET)-based enzymatic activity assay was employed. This assay was conducted sequentially, beginning with a preliminary screening of crude extracts, followed by dose-response validation of the pure monomeric compounds.

For the preliminary screening of crude extracts, the purified recombinant Mpro (final concentration 2 μmol/L) was co-incubated with various concentrations of the aqueous or ethanol extract of *Siphonostegia chinensis* Benth. in the assay buffer at room temperature for 15 min. Subsequently, the enzymatic reaction was initiated by adding a FRET substrate probe based on the Dabcyl-KTSAVLQSGFRKME-Edans sequence (final concentration 2 μmol/L). The reaction progress was monitored in real-time using a fluorescence microplate reader with an excitation wavelength of 437 nm. The emission fluorescence intensities at 570 nm and 632 nm were simultaneously recorded. The ratio of these fluorescence intensities (RFU570/632) was used to dynamically reflect the extent of substrate cleavage, where a higher ratio indicates greater residual Mpro enzymatic activity. A positive control group containing only the enzyme and substrate (representing 100% enzyme activity) and a background control group containing only the substrate without the enzyme were included. The relative inhibitory effect of the crude extracts was evaluated by comparing the plateau-phase RFU570/632 ratios.

Based on the results from the crude extract screening and the subsequent candidate compounds identified via BLI fishing and mass spectrometry, the inhibitory activity of the pure compounds (verbascoside and 3,4-dicaffeoylquinic acid) was verified to confirm whether they were the direct active components responsible for the extract’s effect. The verification experiments utilized the same FRET-based detection system. The inhibitory effect of these monomeric compounds on Mpro enzymatic activity was confirmed by comparing the changes in enzyme reaction kinetics before and after the addition of the compounds. GC376 (MedChemExpress, USA) dissolved in DMSO was used as a positive control at a final concentration of 10 µM. All experiments were performed in triplicate, and relative enzyme activity was calculated as (RFU570/632 of sample/RFU570/632 of vehicle control) × 100%.

### 2.4 Bio-layer interferometry (BLI) based ligand fishing and affinity measurement

The interaction between SARS-CoV-2 Mpro and the crude extract or pure compounds from Siphonostegia chinensis Benth. Was analyzed using an OCTET® R8 molecular interaction analysis system (Sartorius AG, Germany) based on bio-layer interferometry. Sample and sensor preparation: Biotinylated Mpro protein was immobilized onto the surface of streptavidin (SA)-coated biosensor probes to achieve directional capture of the target protein. The crude herbal extract samples were centrifuged at 10,000 × g for 5 min, and the supernatants were filtered through a 0.22 μm membrane before use. Pure compounds were prepared as a series of gradient concentrations in the experimental buffer. Binding kinetics and affinity measurement for pure compounds: For the pure compounds, a standard “sandwich method” was used for kinetic analysis. The assay procedure was set as follows: baseline equilibration in buffer for 60 s, followed by immersion of the Mpro-loaded sensor into different concentrations of the compound solution for association (600 s), and finally transfer to a buffer-only solution for dissociation monitoring (120 s). Real-time binding response data were collected using the system’s built-in Octet Analysis Studio 13.0 software (version 13.0). The data were fitted using a 1:1 binding model to calculate the equilibrium dissociation constant (KD). Ligand fishing and enrichment of active components from crude extracts: To capture specific binding components from the complex extract, a “ligand fishing” mode was employed. The procedure was set as follows: after baseline equilibration for 60 s, Mpro protein was loaded onto the sensor surface for 900 s. Following a brief baseline stabilization, the sensor was immersed into the herbal extract solution for incubation (600 s) to allow potential ligands to bind to the immobilized Mpro. Subsequently, the sensor was transferred to a wash buffer for 300 s to remove non-specifically adsorbed impurities. This “binding-wash” cycle could be repeated if necessary to enrich the active components. Finally, the complexes enriched on the sensor surface were either eluted or directly subjected to subsequent mass spectrometric identification.

### 2.5 Mass spectrometric identification of the fished components

Ultra-performance liquid chromatography coupled with quadrupole time-of-flight mass spectrometry (UPLC-QTOF-MS) was employed for identification. The system consisted of an Acquity UHPLC H-Class system (Waters Corp., USA) and a Xevo G2-XS QTof mass spectrometer (Waters Corp., USA) equipped with an electrospray ionization (ESI) source and dynamic background subtraction (DBS) capability. Chromatographic conditions: The separation was performed on an Acquity UPLC® HSS T3 C18 column (2.1 mm × 100 mm, 1.8 μm) maintained at 40 °C. The flow rate was 0.3 mL/min, and the injection volume was 1 μL. The mobile phase consisted of (A) 0.1% formic acid in water and (B) 0.1% formic acid in acetonitrile. A gradient elution program was used: 0–27 min (A: 95% → 0%, B: 5% → 100%). Mass spectrometric conditions: Ionization was performed in negative electrospray ionization (ESI-) mode. The capillary voltage was set at 2.5 kV, the ion source temperature at 140 °C, and the desolvation gas temperature at 450 °C. The cone voltage was 40 V with a cone gas flow of 50 L/h, and the desolvation gas flow was 800 L/h. The collision energy was ramped from 10 to 45 V. The full-scan mass range was *m/z* 50–1200 with a scan time of 0.2 s. Leucine-enkephalin (ESI^−^: *m/z* 554.2615) was used as the lock mass calibration compound. Data were acquired using MassLynx v4.1 software in MSE Continuum mode.

### 2.6 Validation of Mpro inhibitory activity for MS-identified compounds

The two compounds identified by mass spectrometry (verbascoside and 3,4-dicaffeoylquinic acid; see Section 2.5 for MS methodology and Section 3.3.2 for structural confirmation) were subjected to enzymatic inhibition assays to validate their functional activity against Mpro.

Verbascoside and 3,4-dicaffeoylquinic acid (purchased from Shanghai Yuanye Bio-Technology Co., Ltd., China) were accurately weighed and dissolved to prepare 2 mmol/L stock solutions. The experiment was divided into three groups: the experimental group (drug + probe + Mpro), the negative control group (probe + Mpro), and the blank control group (probe only). For the experimental group, Mpro at a final concentration of 2 μmol/L was mixed with 50 μL of the drug solution and incubated at room temperature for 15 min, followed by the addition of the fluorescent probe to a final concentration of 2 μmol/L. The negative control group contained Mpro at a final concentration of 2 μmol/L, incubated at room temperature for 15 min before adding the fluorescent probe (2 μmol/L final concentration). The blank control group received only the fluorescent probe at a final concentration of 2 μmol/L. The total volume in each well was 100 μL. After thorough mixing, the signal was detected using a fluorescence microplate reader with an excitation wavelength of 437 nm (characteristic absorption peak of the probe) and emission wavelengths of 570 nm and 632 nm to record the relative fluorescence units (RFU).

### 2.7 Molecular docking and molecular dynamics simulations

To investigate the potential binding modes and interactions of the active compounds with SARS-CoV-2 Mpro, molecular docking studies were performed. The receptor protein structure was retrieved from the Protein Data Bank (PDB ID: 7VH8, 1.59Å resolution). Pre-processing involved removing water molecules, the original ligand, and irrelevant ions, followed by adding hydrogen atoms and assigning Gasteiger charges. The 3D structures of the active compounds, verbascoside and 3,4-dicaffeoylquinic acid, were obtained from the PubChem database and geometrically optimized using the MMFF94 force field.

Docking calculations were carried out using AutoDock Vina software. The receptor protein was prepared by removing water molecules, the original ligand, and irrelevant ions, followed by adding polar hydrogens and assigning Gasteiger charges. The grid box for docking was defined using UCSF Chimera to encompass the entire Mpro catalytic pocket, with the center positioned at the geometric center of the active site residues (referenced to CYS145 and HIS41). The grid box dimensions were set to 35.0814 Å × 31.9215 Å × 39.7658 Å with a grid point spacing of 1.0 Å, covering the catalytic dyad (HIS41-CYS145) and surrounding substrate-binding subsites (S1, S2, and S4). The precise grid center coordinates were X: -13.4462, Y: 17.0797, Z: -35.1758 (based on the PDB structure 7VH8 after alignment). Exhaustiveness was set to 20 to enhance sampling, and up to 10 binding poses were generated for each ligand. The docking results were ranked by binding free energy calculated by AutoDock Vina, and the top-ranked conformation (i.e., the pose with the lowest binding free energy) was selected for visualization and further analysis.

Molecular dynamics (MD) simulations were conducted to evaluate the structural stability and binding characteristics of the two active compounds in a dynamic environment (Abraham et al., 2015; Van Der Spoel et al., 2005). All simulations were performed using the GROMACS 2022.3 software package.

#### 2.7.1 System preparation

Ligand preprocessing was carried out using AmberTools22. The GAFF force field was applied to the small molecules, and Gaussian 16W was used to add hydrogen atoms and calculate RESP charges. The resulting charge data were incorporated into the topology files of the MD system. The protein was described using the AMBER99SB-ILDN force field. The docked complex was placed in a cubic water box with the TIP3P water model, maintaining a minimum distance of 1.2 nm between the protein surface and the box boundary. An appropriate number of Na^+^ ions were added to neutralize the system charge.

#### 2.7.2 Energy minimization and equilibration

Energy minimization was performed using the steepest descent algorithm to eliminate steric clashes. The system was then equilibrated in two stages: 100 ps of NVT equilibration (constant temperature of 300 K, using the V-rescale thermostat with a coupling constant of 0.1 ps), followed by 100 ps of NPT equilibration (constant pressure of 1 bar, using the Berendsen barostat with a coupling constant of 0.1 ps).

#### 2.7.3 Production MD simulation

A production MD simulation was then run for 100 ns under NPT conditions, consisting of 50,000,000 steps with an integration time step of 2 fs. Van der Waals interactions were treated using a cutoff of 1.2 nm, and long-range electrostatics were handled using the Particle Mesh Ewald (PME) method. Bond lengths were constrained using the LINCS algorithm.

#### 2.7.4 Trajectory analysis

After simulation completion, trajectory analysis was performed using built-in GROMACS tools. The following metrics were calculated: root mean square deviation (RMSD) of the protein backbone, root mean square fluctuation (RMSF) of individual residues, radius of gyration (Rg), solvent accessible surface area (SASA), and number of hydrogen bonds.

#### 2.7.5 Binding free energy calculations

The binding free energies between each ligand and Mpro were calculated using the MM/GBSA (Molecular Mechanics/Generalized Born Surface Area) method. Due to the incompatibility between GROMACS trajectories and AMBER’s MM-PBSA.py, we employed the gmx_MMPBSA tool (version 1.6), which is specifically designed to process GROMACS output files. To ensure the analysis was performed on the equilibrated state of the system, frames from the last 20 ns of the 100 ns simulation trajectory were sampled at 1 ns intervals, yielding 20 frames for analysis. The final binding free energy was calculated as the average over these 20 frames. The calculations were performed using the GB-OBC1 (igb = 5) implicit solvent model with the following parameters: (1) Solute dielectric constant: 1; (2) Solvent dielectric constant: 80; (3) Salt concentration: 0.15 M (to mimic physiological ionic strength); (4) Surface tension: 0.0072 kcal/(mol·Å^2^); (5) Temperature: 300 K.

The binding free energy was decomposed into individual contributions, including van der Waals (ΔVDWAALS), electrostatic (ΔEelec), polar solvation (ΔEGB), and non-polar solvation (ΔEsurf) energies. Gas-phase energy (ΔGgas) was calculated as the sum of ΔVDWAALS and ΔEelec, while total solvation energy (ΔGsolvation) was the sum of ΔEGB and ΔEsurf. The overall binding free energy (ΔTotal) was obtained by combining gas-phase and solvation contributions. All energy components were calculated for each complex to quantitatively assess the thermodynamic driving forces underlying ligand binding.

### 2.8 *In silico* ADMET prediction and evaluation of drug-likeness

The absorption, distribution, metabolism, and excretion (ADME) properties of the compounds were predicted *in silico* using the SwissADME online server. Toxicity was predicted using the organ toxicity and genotoxicity models within the admetSAR 3.0 tool, as described in the literature. The assessed organ toxicity endpoints were categorized as follows: drug-induced liver injury (DILI), inhibition of the human ether-a-go-go-related gene (hERG) channel, acute toxicity, and eye damage/irritation. The Simplified Molecular Input Line Entry System (SMILES) notations of the two selected compounds were used as the starting point and input data for the SwissADME and admetSAR 3.0 web servers to conduct the ADME and toxicity predictions. Drug-likeness was evaluated based on Lipinski’s rule of five.

### 2.9 Statistical analysis

Statistical analysis and graph generation were performed using GraphPad Prism 7.0 software. Data are presented as the mean ± standard deviation.

## 3 Results

### 3.1 Inhibitory activity of *Siphonostegia chinensis* Benth. crude extracts against Mpro

*Siphonostegia chinensis* Benth. (BLJN), a traditional Chinese medicine preparation used for treating upper respiratory tract infections such as pharyngitis, has shown therapeutic potential for COVID-19-related complications (Ning, et al., 2021).

To evaluate the inhibitory effects of different extracts of BLJN on Mpro activity, a fluorescence resonance energy transfer (FRET) probe was employed to monitor the enzyme-substrate interaction. The results demonstrated that the presence of both extracts led to a dose-dependent decrease in the fluorescent signal, indicating significant inhibition of the enzyme-substrate interaction. The shift in the emission spectrum was consistent with conformational changes in the enzyme upon inhibitor binding, suggesting that the compounds effectively blocked the active site or disrupted the formation of the enzyme-substrate complex. These findings provide further insight into the inhibitory mechanism, confirming that the extract acts as an effective Mpro inhibitor through specific binding interactions.

Furthermore, differences in Mpro inhibition were observed between the two extracts. The Michaelis-Menten curve for the test group treated with 20 mg/mL of the ethanol extract was lower and flatter compared to that of the group treated with the same concentration of the aqueous extract, and more closely resembled the curve of the blank group. This suggests that at the same concentration, the ethanol extract more effectively inhibits Mpro activity. At a concentration of 200 mg/mL, both extracts completely inhibited Mpro, whereas no inhibitory activity was observed at 2 mg/mL (Supplementary Figure S2). These results indicate that the ethanol extract contains a higher concentration of the inhibitory component (s).

To further analyze the binding interactions between the two extracts and Mpro, bio-layer interferometry (BLI) was used for real-time binding monitoring. The results showed that both extracts exhibited a dose-dependent binding response, and the response curves displayed a clear saturation trend, indicating the presence of active components capable of specific interactions with Mpro. The binding kinetic characteristics further suggested that the active components present in the extracts possess a certain affinity for Mpro. This may be related to the enzyme inhibitory activity observed *in vitro* and provides a basis for the subsequent identification of specific active compounds using the ligand fishing strategy.

### 3.2 BLI-based ligand fishing and binding characterization of active components

To specifically capture the active components interacting with SARS-CoV-2 Mpro from the complex extract of *Siphonostegia chinensis* Benth., bio-layer interferometry (BLI) was employed for ligand fishing and real-time binding analysis. As shown in Supplementary Figure S3, streptavidin (SA)-coated biosensors were used. The recombinant Mpro protein was immobilized onto the sensor tips via a biotin-streptavidin system, forming a stable target biomolecular layer. Subsequently, the sensors were immersed into solutions of the BLJN extracts at different concentrations (25, 50, 100, 150, 200, and 250 mg/mL, based on crude drug equivalent) to monitor the association and dissociation processes between components in the extracts and the immobilized Mpro in real-time.

The BLI sensor response curves (sensorgrams) clearly revealed the binding kinetic characteristics and significant differences between the two extracts (Supplementary Figures S3A,B). At the same crude drug concentration, the ethanol extract exhibited a markedly stronger binding response signal, and its dissociation rate was significantly slower than that of the aqueous extract. This indicates that the components in the ethanol extract binding to Mpro are not only more abundant but also form more stable complexes. In contrast, the aqueous extract showed a weak binding response and rapid dissociation. This result clearly demonstrates that the ethanol extract is enriched with active components that bind to Mpro with high affinity, whereas such components are present in very low amounts or possess insufficient affinity in the aqueous extract. Following the BLI enrichment and completion of the association-dissociation cycle, the eluate from the sensor surface (or the sensor tip with bound compounds directly processed) was collected for UPLC-Q-TOF-MS analysis to identify the chemical components specifically captured by Mpro.

### 3.3 Identification and comparison of the fished components by UPLC-Q-TOF-MS

#### 3.3.1 Identification of potential active components fished by Mpro via UPLC-Q-TOF-MS

Under the optimized UPLC-Q-TOF-MS/MS conditions, structural identification of the enriched active ligands was performed. Using the molecular fishing strategy, a batch of small-molecule compounds with high binding affinity to Mpro was successfully captured from the different extracts of *Siphonostegia chinensis* Benth. The total ion chromatogram (TIC), quasi-molecular ion peaks, and characteristic secondary fragment information for each compound were obtained (as shown in Supplementary Figures S4A,B). Based on the comparison of retention time, accurate mass, typical neutral loss patterns, and characteristic fragmentation rules, combined with data from reference standards, databases (e.g., UNIFI/GNPS), and literature, a total of 28 potential active compounds were tentatively identified (see Supplementary Tables S1, S2). Among them, 9 compounds binding to Mpro were identified in the aqueous extract, including 5 flavonoids, 1 anthraquinone, 2 water-soluble phenolic acids, and 1 phenylpropanoid. In the ethanol extract, 28 compounds were identified, specifically including 7 flavonoids, 5 terpenoids, 4 phenylpropanoids, 5 phenolic acids and their derivatives, 3 aromatic compounds, 2 fatty acids, and 2 other types of compounds.

#### 3.3.2 Structural confirmation based on MS/MS fragment ions

To evaluate the potential binding capacity of the identified compounds to SARS-CoV-2 Mpro, molecular docking studies were conducted. The results showed that among the preliminarily identified 28 constituents, verbascoside and 3,4-dicaffeoylquinic acid had the lowest docking scores (−8.4 kcal/mol and −8.1 kcal/mol, respectively) (Supplementary Table S3). They formed stable binding with key residues in the Mpro catalytic pocket through hydrogen bonds and hydrophobic interactions, suggesting their binding modes are reasonable and potentially inhibitory.

**FIGURE 2:**
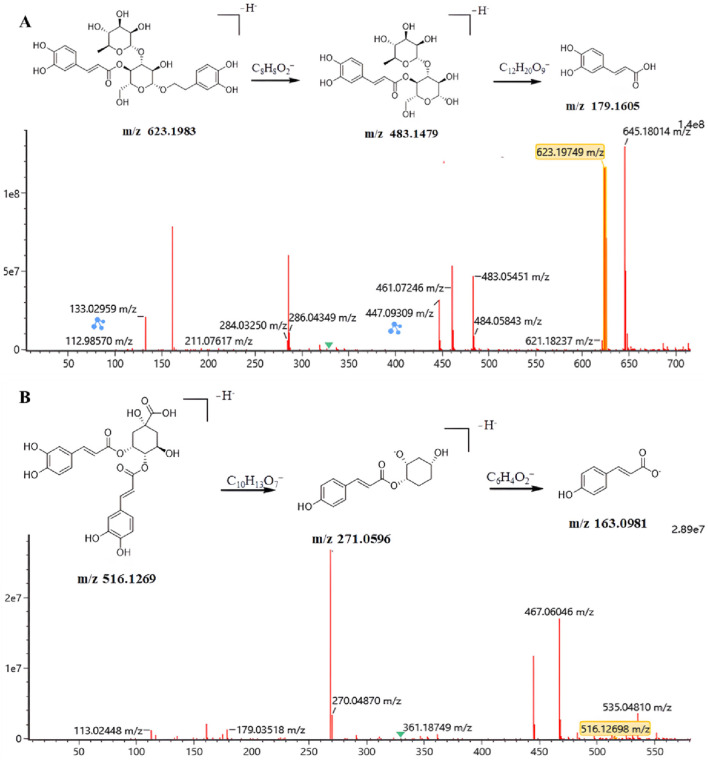
MS/MS fragmentation spectra of the two lead compounds identified via BLI fishing and UPLC-ESI-QTOF-MS/MS. **(A)** Verbascoside and **(B)** 3,4-dicaffeoylquinic acid. Characteristic fragment ions are annotated, supporting the structural identification of the compounds specifically captured by Mpro.

Subsequently, a standard reference comparison method was employed. By comparing the retention times and secondary mass spectrometry fragmentation patterns of the target compounds with those of authentic standards under identical chromatographic-mass spectrometric conditions, verbascoside and 3,4-dicaffeoylquinic acid were unambiguously identified. For verbascoside, its chromatographic peak exhibited a consistent retention time with the standard on UPLC. In negative ion mode, the quasi-molecular ion peak [M-H]^-^ was observed at *m/z* 623.1983 (see Figure 2A). The major fragment ions included m/z 483.1479, resulting from the loss of a caffeoyl moiety (−140 Da), and m/z 179.1605 from the further loss of a glycoside unit. These fragments showed high consistency with the mass spectrum of the standard, confirming the compound as verbascoside (see Supplementary Figure S5C).

3,4-Dicaffeoylquinic acid was also confirmed by comparison with the authentic standard. In negative ion mode, the quasi-molecular ion peak [M-H]^-^ was observed at *m/z* 516.1163. Its MS/MS spectrum (see Figure 2B) displayed the characteristic fragmentation pattern of two caffeoyl groups. The major fragment ions at *m/z* 179 and 163 corresponded to the caffeoyl cation and the characteristic ion of the quinic acid core skeleton, respectively, which completely matched the spectrum of the standard, confirming the compound as 3,4-dicaffeoylquinic acid (Supplementary Figure S5D).

### 3.4 BLI verification of the binding affinity between the active compounds and Mpro

**FIGURE 3:**
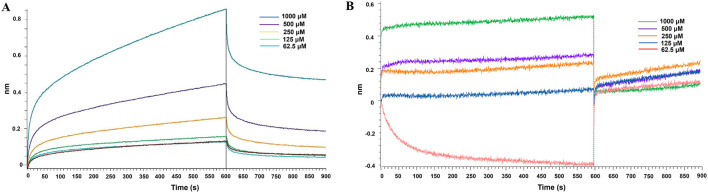
BLI sensorgrams for the binding of purified compounds to Mpro. **(A)** Verbascoside; **(B)** 3,4-dicaffeoylquinic acid. Sensorgrams show real-time association and dissociation at different compound concentrations. The equilibrium dissociation constant (KD) was derived from a 1:1 binding fit.

To further confirm the specific interactions of verbascoside and 3,4-dicaffeoylquinic acid with Mpro, their binding kinetics were determined using bio-layer interferometry (BLI). The BLI sensorgrams clearly displayed the real-time association and dissociation processes between these two compounds and the immobilized Mpro (Figure 3). Verbascoside exhibited a rapid association response and a relatively slow dissociation trend across concentration gradients (Figure 3A), indicating the formation of a relatively stable complex with Mpro. In contrast, although 3,4-dicaffeoylquinic acid also showed concentration-dependent binding, its dissociation rate was significantly faster (Figure 3B).

By fitting the sensor data to a 1:1 binding model, quantitative affinity parameters were obtained (Table 1). The equilibrium dissociation constant (KD) for verbascoside binding to Mpro was 2.149 × 10^−6^ M, with a dissociation rate constant (k_diss) of 1.130 × 10^−3^ s^-1^. For 3,4-dicaffeoylquinic acid, the KD value was 2.487 × 10^−5^ M, with a kdiss of 7.807 × 10^−2^ s^-1^. These data indicate that verbascoside possesses a higher affinity for Mpro than 3,4-dicaffeoylquinic acid and forms a more stable complex. This is consistent with its more favorable docking score in the molecular docking studies. The results further verify that both verbascoside and 3,4-dicaffeoylquinic acid can bind specifically to SARS-CoV-2 Mpro in a dose-dependent manner, providing physical interaction support for their feasibility as potential Mpro inhibitors.

**TABLE 1:** Binding affinity of lead compounds for Mpro determined by BLI.

TCM	*K*_*D*_ (mol/L)	*K*_*dis*_ (1/s)
Verbascoside	2.149 × 10^−6^	1.130 × 10^−3^
3,4-dicaffeoylquinic acid	2.487 × 10^−5^	7.807 × 10^−2^

### 3.5 Validation of the inhibitory activity of monomeric compounds against Mpro

**FIGURE 4:**
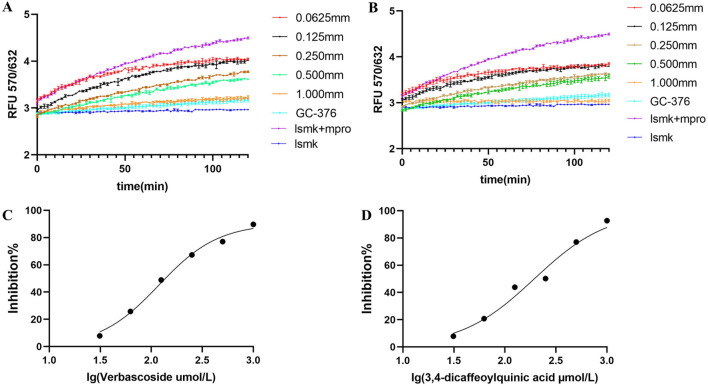
Inhibitory effects of purified compounds on Mpro enzymatic activity. **(A)** Verbascoside; **(B)** 3,4-dicaffeoylquinic acid. Dose-response curves showing the inhibition of Mpro activity by the compounds. GC376 was used as a positive control. Semi-logarithmic plots of the inhibition data for verbascoside **(C)** and 3,4-dicaffeoylquinic acid **(D)** used to determine the half-maximal inhibitory concentration (IC50). The fitted IC50 values are 0.076 µM and 0.194 µM, respectively.

To verify whether verbascoside and 3,4-dicaffeoylquinic acid possess the function of inhibiting Mpro enzymatic activity, the same FRET-based detection system used for crude extract screening was employed. The positive control GC376 confirmed the validity of the FRET-based assay system. The change in fluorescent signal during Mpro cleavage of the substrate probe in the presence of different compound concentrations was monitored to assess their inhibitory potency. The experimental results showed that both compounds significantly inhibited the proteolytic activity of Mpro in a concentration-dependent manner (Figure 4). At the same concentration, the inhibitory effect of verbascoside was markedly stronger than that of 3,4-dicaffeoylquinic acid (Figure 4A), a result consistent with the higher binding affinity of verbascoside observed in the aforementioned BLI experiment (Table 2). Although 3,4-dicaffeoylquinic acid also displayed clear inhibitory activity, a higher concentration was required to achieve a similar level of inhibition (Figure 4B).

**TABLE 2:** MM/GBSA binding free energy decomposition for the Mpro complexes.

Contribution components	Verbascoside	3,4-dicaffeoylquinic acid
Δ_VDWAALS_	−45.88 ± 0.06	−41.99 ± 0.64
ΔE_elec_	−25.86 ± 4.21	−50.19 ± 4.44
ΔE_GB_	40.23 ± 1.17	58.53 ± 0.96
ΔE_surf_	−6.49 ± 0.20	−6.07 ± 0.05
ΔG_gas_	−71.74 ± 4.21	−92.18 ± 4.48
ΔG_solvation_	33.74 ± 1.19	52.46 ± 0.96
ΔTotal	−37.99 ± 4.37	−39.72 ± 1.59

Further nonlinear fitting of the dose-response curves calculated the half-maximal inhibitory concentration (IC_50_) values. The IC_50_ of verbascoside and 3,4-dicaffeoylquinic acid against Mpro were 0.076 µM and 0.194 µM, respectively (see Figures 4C,D). This quantitative data further functionally confirms that verbascoside has stronger Mpro inhibitory potency, which aligns with the order of BLI binding affinity and preliminary inhibitory activity.

### 3.6 Molecular dynamics analysis of the compound-Mpro binding

To gain deeper insights into the interaction mechanisms between the lead compounds and Mpro, we first analyzed their optimal docking conformations and further evaluated the stability of the complexes in a dynamic environment through molecular dynamics (MD) simulations.

**FIGURE 5:**
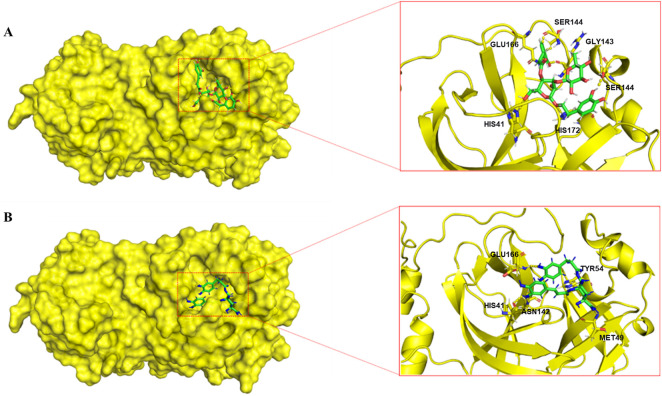
Molecular docking poses of the lead compounds within the Mpro binding pocket. **(A)** Verbascoside (docking score: −8.4 kcal/mol); **(B)** 3,4-dicaffeoylquinic acid (docking score: −8.1 kcal/mol). Key interacting residues (e.g., His41, Cys145, Glu166) are shown.

Analysis of the optimal docking conformations (Figure 5) revealed that both compounds successfully embedded into the hydrophobic catalytic pocket formed by key residues (e.g., HIS41, CYS145, MET165, and GLU166) and established close-range van der Waals interactions with the catalytic dyad HIS41-CYS145. Verbascoside formed extensive hydrophobic contacts with the S1/S2 subsites (e.g., MET165, HIS172) via its phenylethanoid glycoside core. Concurrently, multiple hydroxyl groups on its glucosyl moiety established a stable hydrogen bond network with the backbone atoms of GLY143 and SER144, as well as the side chain of GLU166 in Mpro. The two caffeoyl groups of 3,4-dicaffeoylquinic acid extended into a hydrophobic region surrounded by MET49 and TYR54, while the core quinic acid moiety formed polar contacts with HIS41 and ASN142. Its carboxyl group also formed a crucial hydrogen bond with GLU166. Notably, both compounds bound within the Mpro catalytic pocket exclusively through non-covalent interactions (including hydrogen bonds and hydrophobic contacts), with no proximity or orientation conducive to covalent bond formation with the catalytic CYS145. This binding mode is characteristic of reversible, non-covalent inhibitors and provides a structural basis for understanding their inhibitory mechanisms.

**FIGURE 6:**
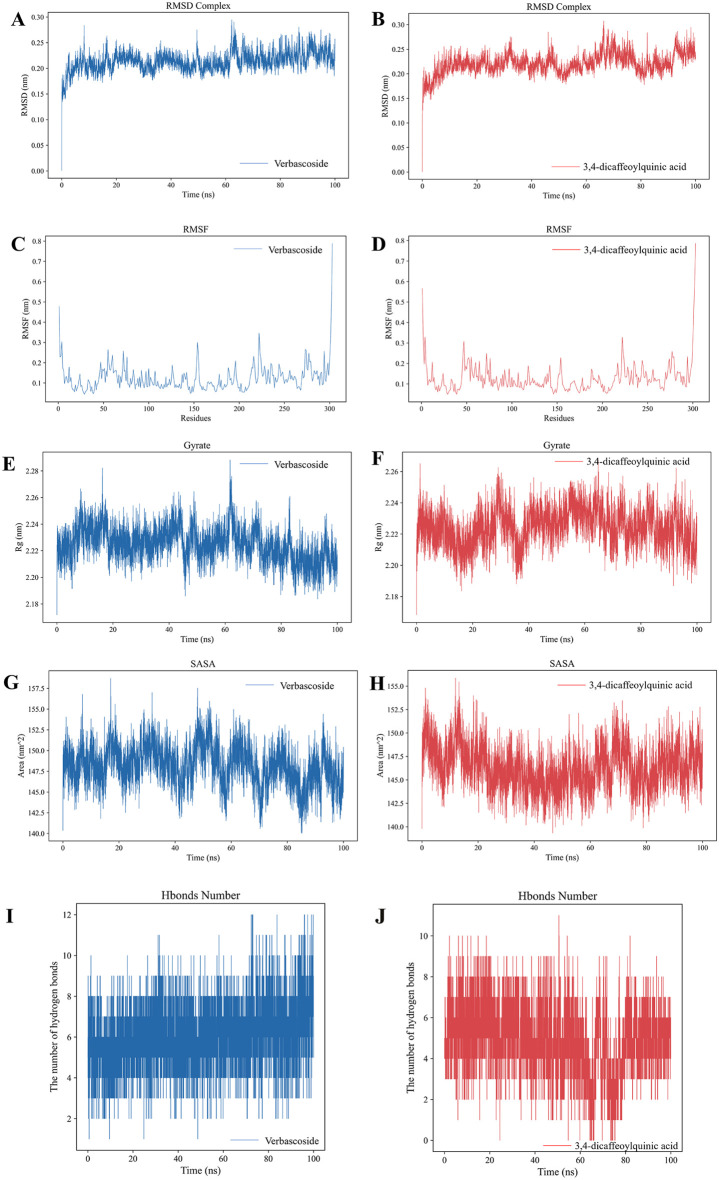
Molecular dynamics simulation analysis of the Mpro-compound complexes. **(A,B)** Root-mean-square deviation (RMSD) of the protein backbone for the complexes with verbascoside and 3,4-dicaffeoylquinic acid, respectively. **(C,D)** Root-mean-square fluctuation (RMSF) of protein residues for the respective complexes. **(E,F)** RMSD of the ligand heavy atoms. **(G,H)** Solvent-accessible surface area (SASA) of the complexes. **(I,J)** Number of intermolecular hydrogen bonds between Mpro and the ligand over the simulation time.

To further assess the structural stability of the complexes under dynamic conditions in an aqueous environment, 100 ns MD simulations were performed for Mpro complexed with each compound. Analysis of the simulation trajectories indicated that both complex systems exhibited good structural stability (Figure 6). During the equilibrium phase, the root mean square deviation (RMSD) of the Mpro protein backbone remained stable (verbascoside complex: 0.21 ± 0.02 nm; 3,4-dicaffeoylquinic acid complex: 0.22 ± 0.02 nm). Residues near the active site showed minor fluctuations, indicating that ligand binding did not significantly perturb the protein’s catalytic core.

Further interaction analysis (Figures 6I,J) showed that verbascoside formed an average of approximately 6 stable hydrogen bonds with Mpro, while 3,4-dicaffeoylquinic acid formed an average of about 5 hydrogen bonds. MM/GBSA binding free energy calculations (Supplementary Table S4) revealed that the total binding free energy (ΔG_total) was negative for both complexes (verbascoside: -37.99 ± 4.37 kcal/mol; 3,4-dicaffeoylquinic acid: -39.72 ± 1.59 kcal/mol), confirming the spontaneity of their binding (Miltner, et al., 2023). Energy decomposition analysis further elucidated the differences in their binding driving forces: the binding of verbascoside relied mainly on a balanced contribution from van der Waals and electrostatic interactions, whereas 3,4-dicaffeoylquinic acid exhibited stronger electrostatic interactions, which were, however, substantially offset by polar solvation energy.

### 3.7 Predictive analysis and comparison of ADMET properties

To preliminarily assess the drug-likeness and safety profiles of the two lead compounds, their absorption, distribution, metabolism, excretion, and toxicity (ADMET) properties were systematically predicted using computational simulations (Supplementary Table S4).

Regarding absorption, the predictions indicated low Caco-2 cell permeability for both compounds: 0.002 cm/s for verbascoside and 0.007 cm/s for 3,4-dicaffeoylquinic acid. Both were also identified as potential substrates of P-glycoprotein (probabilities >0.5), suggesting potential limitations in their intestinal absorption.

In terms of distribution, both compounds showed a low probability of blood-brain barrier penetration (BBB probabilities <0.13). However, they exhibited high predicted plasma protein binding (PPB) rates: 0.641 for verbascoside and 0.839 for 3,4-dicaffeoylquinic acid, which may influence their *in vivo* distribution and efficacy.

Metabolism and toxicity predictions suggested a low risk of inhibiting the major drug-metabolizing enzymes CYP3A4 and CYP2D6 for both compounds. Ames test predictions were negative, indicating a lack of mutagenicity. However, potential safety concerns require attention, particularly the risk of human ether-à-go-go-related gene (hERG) potassium channel inhibition (probability range: 0.291–0.393), which could pose a threat to cardiac safety. Furthermore, verbascoside showed a relatively high risk of drug-induced liver injury (DILI) (probability: 0.554), warranting careful evaluation in subsequent development. Concerning acute oral toxicity, the predicted LD_50_ value for 3,4-dicaffeoylquinic acid (2.315 log mg/kg) was higher than that for verbascoside (1.92 log mg/kg), suggesting a lower acute toxicity for the former.

## 4 Discussion

Integrating the relevant literature on the chemical composition of *Siphonostegia chinensis* Benth. (BLJN) with the findings of this study, it is evident that different extraction methods significantly influence its compound profile, which in turn determines the efficacy of the extracts in inhibiting SARS-CoV-2 Mpro activity. This study found that the ethanol extract exhibited stronger Mpro inhibitory capacity compared to the aqueous extract. This is closely related to the ethanol extraction process being more effective at extracting lipophilic or mid-polarity active components, such as flavonoids and phenolic acids (Pan, et al., 2023; Shawky, et al., 2024). Traditionally, the aqueous extract of BLJN has been primarily used for treating inflammatory conditions like upper respiratory tract infections, where its mechanism likely relies more on the anti-inflammatory and immunomodulatory activities of its water-soluble constituents. In contrast, this study demonstrates that the lipophilic components enriched in the ethanol extract, such as 3,4-dicaffeoylquinic acid, can exert antiviral effects by directly inhibiting the viral Mpro. This provides both chemical and functional rationale for its potential application against SARS-CoV-2.

Regarding dosage form development, the differing characteristics of these extracts allow for the targeted design of distinct drug delivery systems. The bioavailability of the lipophilic components in the ethanol extract could be enhanced through formulation technologies like nanoemulsions, liposomes, or solid dispersions, thereby improving systemic antiviral efficacy. Conversely, the aqueous extract could be further developed into local dosage forms such as sprays, lozenges, or inhalants for direct action on the upper respiratory mucosa to exert anti-inflammatory and immunomodulatory effects. This strategy of matching “extraction method–dosage form–therapeutic effect” offers a promising new direction for the modern formulation development and expansion of indications for BLJN.

The integrated approach of BLI fishing coupled with UPLC-Q-TOF-MS analysis in this study provided direct chemical evidence for the superior activity of the ethanol extract. The identification results revealed that while verbascoside was detected in both extracts, another key active compound—3,4-dicaffeoylquinic acid—was specifically enriched and identified only in the ethanol extract. This finding is significant for several reasons. Firstly, it confirms that the ethanol extraction process more efficiently extracts the lipophilic or mid-polarity components from BLJN that possess high binding affinity for Mpro. Secondly, it suggests that the ethanol extract offers superior diversity and specificity of active components. As a typical phenolic acid, 3,4-dicaffeoylquinic acid contains multiple caffeoyl groups in its structure, conferring relatively strong hydrophobicity. Consequently, its solubility and extraction efficiency are far greater in ethanol than in water. Therefore, the ethanol extract not only retains some common active constituents found in the aqueous extract (e.g., verbascoside) but also additionally enriches specific compounds like 3,4-dicaffeoylquinic acid, which likely contributes significantly to Mpro inhibition. This, from a “chemical constituent-activity relationship” perspective, systematically explains the material basis for the stronger activity exhibited by the ethanol extract in both BLI binding response and enzymatic inhibition assays. It also provides a scientific foundation for the subsequent targeted optimization of extraction processes to enrich specific active ingredients.

The interactions of verbascoside and 3,4-dicaffeoylquinic acid with catalytic residues (e.g., HIS41, MET49 and MET165 in the S2 hydrophobic pocket) and key hydrogen-bonding residues (e.g., GLY143, SER144) of Mpro confirm their binding at the traditional active site. Notably, the strong interaction observed between verbascoside and ARG188 may indicate a unique binding mode capable of penetrating deeper into the S4 pocket, revealing its potential distinction from conventional inhibitors (Evans, et al., 2025). Although the binding affinity of both compounds is in the micromolar range and their interaction with the catalytic CYS145 is limited, their non-covalent inhibitory nature—different from classic covalent inhibitors like nirmatrelvir—still indicates considerable potential for optimization.

This study demonstrates the significant role of verbascoside and 3,4-dicaffeoylquinic acid from *Siphonostegia chinensis* Benth. in inhibiting SARS-CoV-2 Mpro activity, further underscoring the important value of natural compounds in innovative drug discovery. Due to their unique bioactivities and structural diversity, natural compounds have become a valuable source for discovering novel therapeutic agents. Particularly in the context of emerging infectious diseases, screening for active ingredients from natural resources such as traditional Chinese medicine represents an efficient drug discovery strategy. By employing modern analytical techniques (e.g., BLI, UPLC-QTOF-MS) and computational simulations (e.g., molecular docking, dynamics analysis), this study successfully identified active molecules with high binding affinity, showcasing the considerable potential of natural compounds in drug development.

While verbascoside and 3,4-dicaffeoylquinic acid demonstrated promising Mpro inhibitory activity in *in vitro* assays and computational models, their drug-likeness faces challenges. ADMET predictions revealed issues such as low oral permeability and potential P-glycoprotein efflux, which require further optimization (Gao et al., 2021). Despite their current binding affinities, their well-defined mechanism of action and underlying strong binding trends suggest that efficacy could be significantly enhanced through structural optimization. Therefore, future research should focus on rational structural modifications to improve the pharmacokinetic properties and increase the binding affinity of these lead compounds.

## 5 Conclusion

This study successfully established and applied an efficient screening strategy that integrates bio-layer interferometry (BLI) with ultra-performance liquid chromatography coupled to quadrupole time-of-flight mass spectrometry (UPLC-Q-TOF-MS). This approach enabled the rapid and specific identification of two active components targeting the SARS-CoV-2 main protease (Mpro) from the complex extract of the traditional Chinese medicine *Siphonostegia chinensis* Benth.: verbascoside and 3,4-dicaffeoylquinic acid.

Through BLI and kinetic measurements, this study confirmed the specific binding of both components to Mpro, with affinities (KD) in the micromolar range. Further *in vitro* enzymatic inhibition assays validated that both are functional inhibitors: verbascoside exhibited stronger inhibitory activity (IC_50_ = 0.1180 µM), while 3,4-dicaffeoylquinic acid also demonstrated a clear inhibitory effect (IC_50_ = 0.2837 µM). This order of potency is consistent with their respective binding affinities. Molecular docking and dynamics simulations revealed that both compounds bind stably within the catalytic pocket of Mpro via non-covalent interactions, forming key interaction patterns. Preliminary *in silico* ADMET simulations provided directional guidance for the subsequent optimization of these compounds.

In summary, this work not only provides novel natural product-derived candidate compounds for anti-SARS-CoV-2 drug development but also establishes an integrated research paradigm of “BLI screening—MS identification—*in vitro* validation—computational simulation.” This paradigm offers a reliable methodological reference for the efficient and precise mining of protein-targeting bioactive constituents from complex herbal extracts, thereby contributing to the accelerated discovery of innovative drugs based on natural products.

## Data Availability

The original contributions presented in the study are included in the article/Supplementary Material. Further inquiries can be directed to the corresponding author(s).
