# Supplementary material for: SARS-CoV-2 Mpro inhibitors from Siphonostegia chinensis: an integrated biophysical and computational study
Source: Front Chem. 2026 Apr 10;14:1790228. doi: 10.3389/fchem.2026.1790228 (PMC13107242; doi:10.3389/fchem.2026.1790228)
Supplement: Supplementary file 1 [file DataSheet1.docx]

**Supplementary** **Information**

**SARS-CoV-2 Mpro Inhibitors from *Siphonostegia chinensis*: An Integrated Biophysical and Computational Study**

Yingjie Ren^a,b,†^, Wei Wang^d,†^, Dai Zhang^a,b^, Mingliang Zhang^a,b,c^, Hui Zhang ^a,b^, Yali Wu^a,b^, Feiyan Liu^a,b^, Liuqing Yang^a,b^, Pan Wang^a,b^, Lei Chen^e^, Xiaofei Chen ^a,b,✉^, Jinfa Tang ^a,b,c,✉^, Xianqing Ren^a,b,✉^

^a^*Department of Pharmacy, the First Affiliated Hospital of Henan University of Chinese Medicine, Henan Zhengzhou, 450003, China*

^b^*Henan Province Engineering Research Center for Clinical Application, Evaluation and Transformation of Traditional Chinese Medicine, Henan Provincial Key Laboratory for Clinical Pharmacy of Traditional Chinese Medicine, Henan Province Engineering Research Center of Safety Evaluation and Risk Management of Traditional Chinese Medicine, Zhengzhou 450003, Henan, China*

^c^*School of Pharmacy, Henan University of Chinese Medicine, Henan Zhengzhou 450046, China*

^d^*Department of Disease Prevention and Control，the First Affiliated Hospital of Henan University of Chinese Medicine, Henan Zhengzhou, 450003, China*

^e^*Collaborative Innovation Center of Prevention and Treatment of Major Diseases by Chinese and Western Medicine，Henan Province*

^✉^Corresponding authors at: Department of Pharmacy, the First Affiliated Hospital of Henan University of Chinese Medicine, No.19 Renmin Road, JinShui district, Zhengzhou, 450046, China.

^†^These authors have contributed equally to this work and share first authorship.

**Contents**

Figure S1. Expression and purification of SARS-CoV-2 Mpro. SDS-PAGE analysis: Lane M, protein marker; Lane 1, FRET substrate probe; Lane 2, TEV protease; Lane 3, Mpro after TEV cleavage; Lane 4, purified Mpro protein**^……………………………………………………………………………………….^**3

Figure S2. Inhibition of SARS-CoV-2 Mpro by aqueous and ethanol extracts of Siphonostegia chinensis Benth. (A) Aqueous extract; (B) Ethanol extract. The enzymatic activity was monitored by the RFU ratio (570/632 nm) over time. The extracts were tested at different concentrations (based on raw herb weight). “Ismk + Mpro” represents the positive control (enzyme + substrate), and “Ismk” represents the substrate-only background control.**^…………………………………………………………^** 3

Figure S3. BLI sensorgrams for the interaction between SARS-CoV-2 Mpro and crude extracts.(A) Aqueous extract; (B) Ethanol extract. Real-time binding responses at varying concentrations of extracts are shown. The affinity was calculated by fitting to a 1:1 binding model**^………………………^4**

Figure S4. UPLC-Q-TOF-MS total ion chromatograms of the compounds fished from Siphonostegia chinensis extracts by Mpro via BLI. (A) Aqueous extract; (B) Ethanol extract. The chromatograms display the ligands specifically captured and enriched from the complex extracts by BLI-based fishing using immobilized Mpro, followed by UPLC-Q-TOF-MS analysis. The ethanol extract yielded a greater number of detectable compounds, consistent with its higher binding affinity observed in BLI kinetic studies. **^……………………………………………………………………………………………………^5**

Figure S5. Representative chromatograms for the identification of target compounds. (C) Extracted ion chromatogram (EIC) of verbascoside in negative ion mode. (D) Extracted ion chromatogram (EIC) of 3,4-dicaffeoylquinic acid in negative ion mode. **^……………………………………………………………^6**

Table S1. Compounds identified from the aqueous extract by UPLC-Q-TOF-MS after BLI fishing**^………………………………………………………………………………………………………………………………………………^7**

Table S2. Compounds identified from the ethanol extract by UPLC-Q-TOF-MS after BLI fishing**^…^8**

Table S3. Molecular docking scores of identified compounds against SARS-CoV-2 Mpro. **^…………^11**

Table S4. Predicted ADMET properties of the lead compounds. **^…………………………………………………^12**


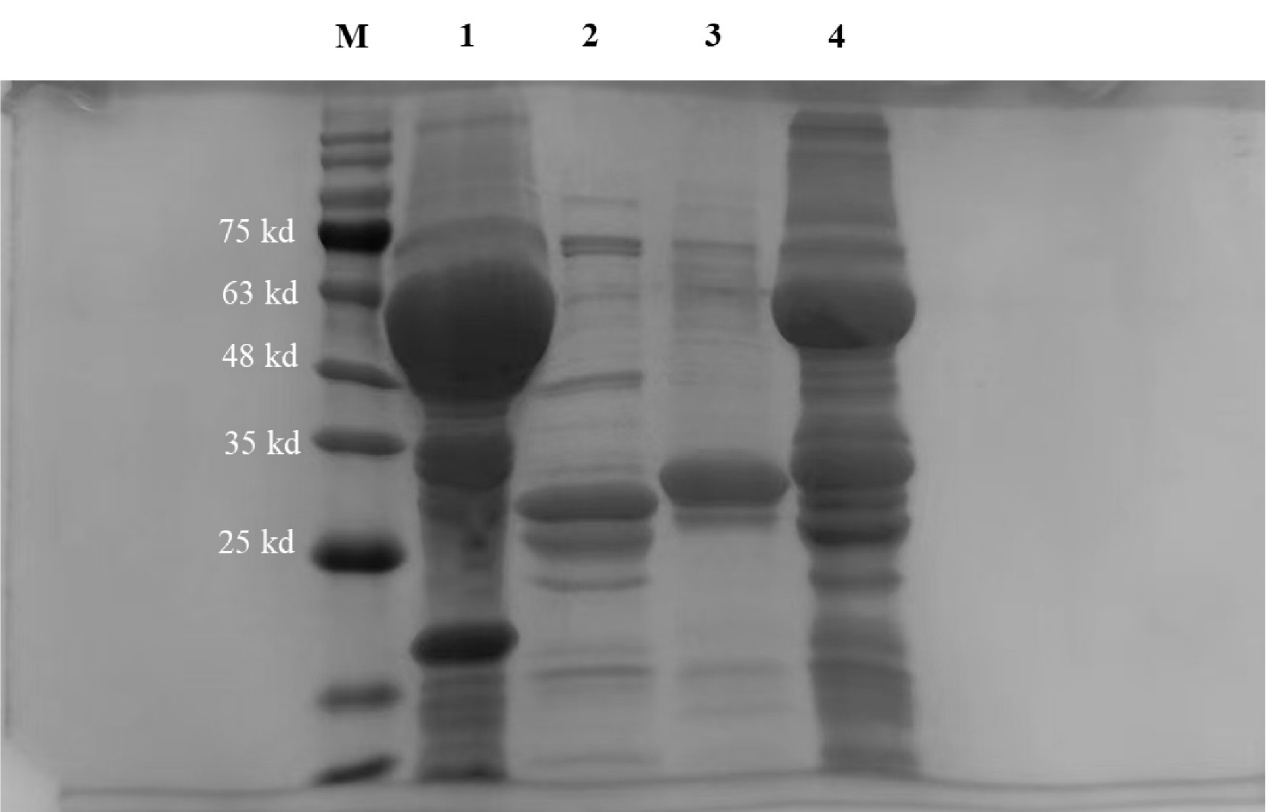


Figure S1. Expression and purification of SARS-CoV-2 Mpro. SDS-PAGE analysis: Lane M, protein marker; Lane 1, FRET substrate probe; Lane 2, TEV protease; Lane 3, Mpro after TEV cleavage; Lane 4, purified Mpro protein


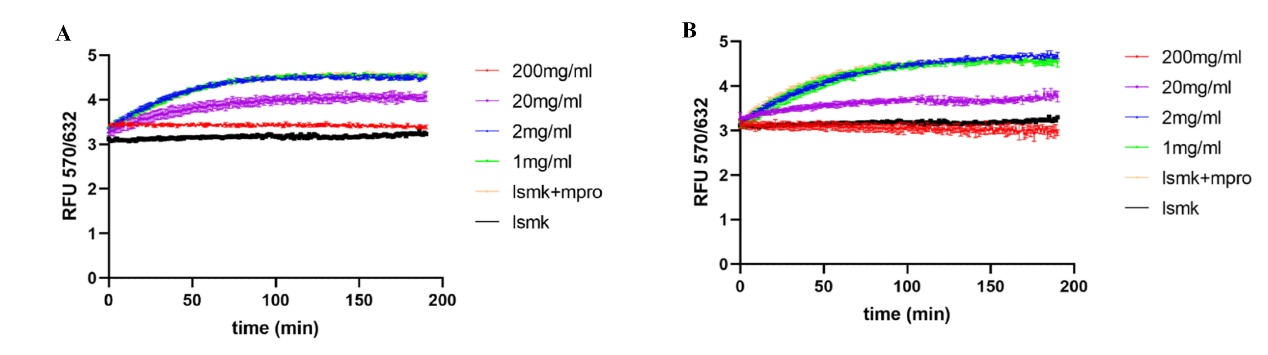


Figure S2. Inhibition of SARS-CoV-2 Mpro by aqueous and ethanol extracts of Siphonostegia chinensis Benth. (A) Aqueous extract; (B) Ethanol extract. The enzymatic activity was monitored by the RFU ratio (570/632 nm) over time. The extracts were tested at different concentrations (based on raw herb weight). “Ismk + Mpro” represents the positive control (enzyme + substrate), and “Ismk” represents the substrate-only background control.


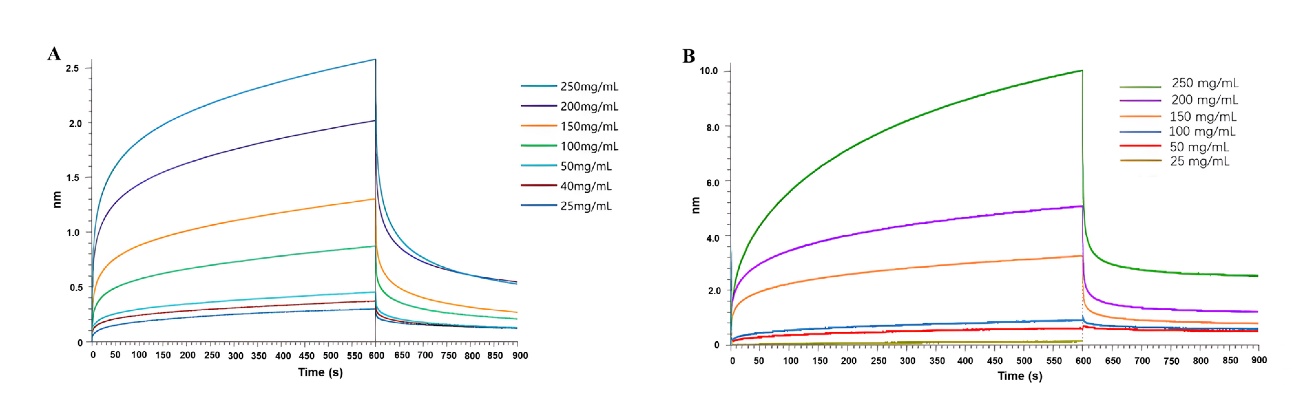


Figure S3. BLI sensorgrams for the interaction between SARS-CoV-2 Mpro and crude extracts.(A) Aqueous extract; (B) Ethanol extract. Real-time binding responses at varying concentrations of extracts are shown. The affinity was calculated by fitting to a 1:1 binding model.


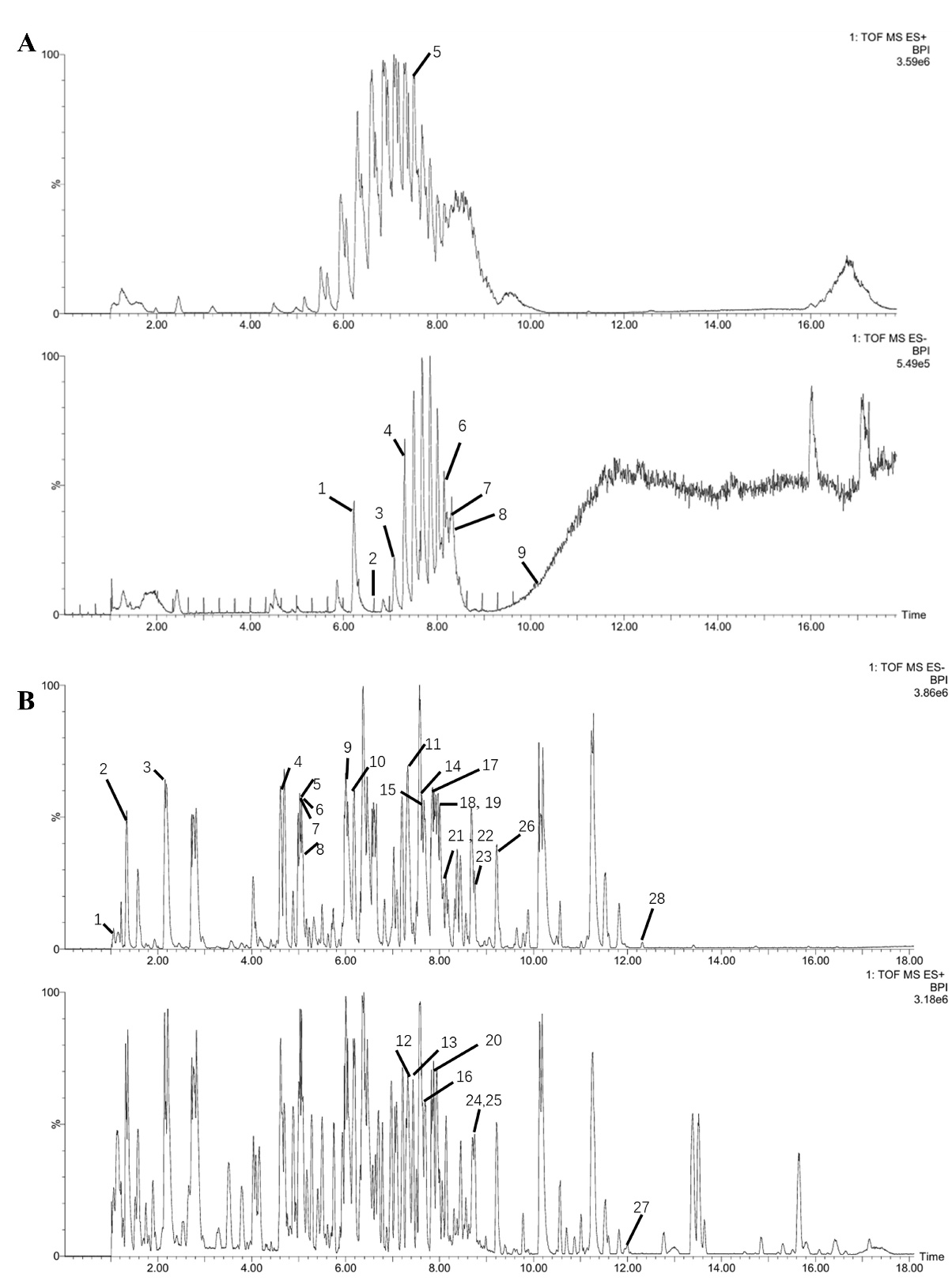


Figure S4. UPLC-Q-TOF-MS total ion chromatograms of the compounds fished from Siphonostegia chinensis extracts by Mpro via BLI. (A) Aqueous extract; (B) Ethanol extract. The chromatograms display the ligands specifically captured and enriched from the complex extracts by BLI-based fishing using immobilized Mpro, followed by UPLC-Q-TOF-MS analysis. The ethanol extract yielded a greater number of detectable compounds, consistent with its higher binding affinity observed in BLI kinetic studies.


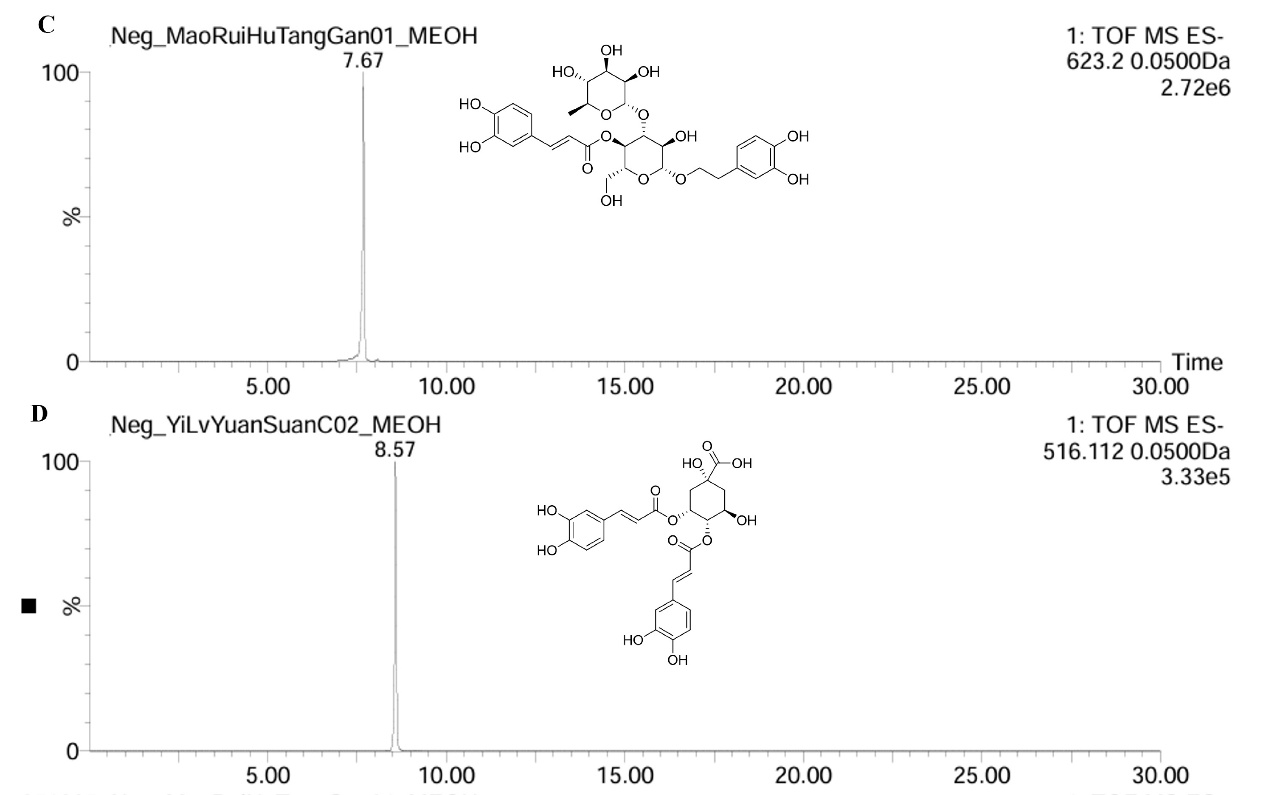


Figure S5. Representative chromatograms for the identification of target compounds. (C) Extracted ion chromatogram (EIC) of verbascoside in negative ion mode. (D) Extracted ion chromatogram (EIC) of 3,4-dicaffeoylquinic acid in negative ion mode.

Table S1. Compounds identified from the aqueous extract by UPLC-Q-TOF-MS after BLI fishing

| No. | Compound Name | *t*_R_(min) | MolecularFormula | Molecular Mass | Chemical Structure |
| --- | --- | --- | --- | --- | --- |
| 1 | Nicotiflorin | 6.09 | C_27_H_30_O_15_ | 593.1529 [M - H]^−^ |  |
| 2 | Sutchuenoside A | 6.56 | C_29_H_32_O_15_ | 619.1667 [M - H]^−^ |  |
| 3 | trans-4-Hydroxycinnamic acid | 7.06 | C_9_H_8_O_3_ | 163.0399 [M - H]^−^ |  |
| 4 | Astragalin | 7.48 | C_21_H_20_O_11_ | 447.094 [M - H]^−^ |  |
| 5 | Verbascoside | 7.74 | C_29_H_36_O_15_ | 669.2034 [M + HCOO]^+^ |  |
| 6 | Rhein-8-glucoside | 8.24 | C_21_H_18_O_11_ | 445.0782 [M - H]^−^ |  |
| 7 | crenatoside | 8.44 | C_29_H_34_O_15_ | 621.1827 [M - H]^−^ |  |
| 8 | Luteolin-7-O-glucuronide | 8.51 | C_21_H_18_O_12_ | 461.0744 [M - H]^−^ |  |
| 9 | Apigenin | 10.9 | C_15_H_10_O_5_ | 269.0461 [M - H]^−^ |  |

Table S2. Compounds identified from the ethanol extract by UPLC-Q-TOF-MS after BLI fishing.

| No. | Compound Name | *t*_R_ (min) | MolecularFormula | Molecular Mass | Chemical Structure |
| --- | --- | --- | --- | --- | --- |
| 1 | p-Anisaldehyde | 1.34 | C_8_H_8_O_2_ | 137.0585 [M + H]^+^ |  |
| 2 | Nootkatone | 1.58 | C_15_H_22_O | 241.1540 [M + Na]^+^ |  |
| 3 | Caproic acid | 2.67 | C_6_H_12_O_2_ | 139.0740 [M + Na]^+^ |  |
| 4 | Isocantleyine | 4.60 | C_11_H_13_NO_3_ | 208.0961 [M + H]^+^ |  |
| 5 | 7-Methoxycoumarin | 5.05 | C_10_H_8_O_3_ | 177.0539 [M + H]^+^ |  |
| 6 | 5,3'-Dihydroxy-6,7,4'-trimethoxy flavanone | 5.05 | C_18_H_18_O_7_ | 405.1200 [M + Li]^+^ |  |
| 7 | (*R*)-3-phenoxy-1,2-propanediol | 5.05 | C_9_H_12_O_3_ | 191.0696 [M + Na]^+^ |  |
| 8 | Isoferulic acid | 5.06 | C_10_H_10_O_4_ | 195.0645 [M + H]^+^ |  |
| 9 | Eupatorin | 6.02 | C_18_H_16_O_7_ | 351.1045 [M + Li]^+^ |  |
| 10 | Eleutheroside B | 6.38 | C_17_H_24_O_9_ | 373.1491 [M + H]^+^ |  |
| 11 | Macranthoin F | 7.20 | C_26_H_26_O_12_ | 531.1476 [M + H]^+^ |  |
| 12 | Apiin | 7.32 | C_26_H_28_O_14_ | 563.1385 [M - H]^−^ |  |
| 13 | 2,3-Dihydrobenzofuran | 7.32 | C_8_H_8_O | 119.0503 [M - H]^−^ |  |
| 14 | Umbelliferone | 7.59 | C_9_H_6_O_3_ | 163.0382 [M + H]^+^ |  |
| 15 | Caffeic acid | 7.59 | C_9_H_8_O_4_ | 181.0486 [M + H]^+^ |  |
| 16 | Verbascoside | 7.67 | C_29_H_36_O_15_ | 623.1983 [M - H] ^−^ |  |
| 17 | Luteolin-7-O-glucuronide | 7.7 | C_21_H_18_O_12_ | 463.0868 [M + H]^+^ |  |
| 18 | Crenatoside | 7.95 | C_29_H_34_O_15_ | 645.1796 [M + Na]^+^ |  |
| 19 | Cynaroside | 7.95 | C_21_H_20_O_11_ | 447.0933 [M - H]^−^ |  |
| 20 | Daucosterol | 7.95 | C_35_H_60_O_6_ | 621.4396 [M+HCOO] ^−^ |  |
| 21 | Loliolid | 8.33 | C_11_H_16_O_3_ | 219.0983 [M + Na]^+^ |  |
| 22 | 3,4-dicaffeoylquinic acid | 8.57 | C_25_H_24_O_12_ | 516.1163 [M + H]^+^ |  |
| 23 | luteolin | 8.71 | C_15_H_10_O_6_ | 287.0541 [M + H]^+^ |  |
| 24 | Rubinaphthin A | 8.75 | C_17_H_18_O_9_ | 411.0917 [M + HCOO] ^−^ |  |
| 25 | 6-Methyl-3,5-heptadien-2-one | 8.75 | C_8_H_12_O | 183.1025 [M + CH_3_COO] ^−^ |  |
| 26 | 4-tert-Butylcatechol | 8.99 | C_10_H_14_O_2_ | 167.1060 [M + H]^+^ |  |
| 27 | Ermanin | 12.18 | C_17_H_14_O_6_ | 359.077 [M+HCOO]^−^ |  |
| 28 | (S)-4,4,7a-Trimethyl-5,6,7,7a-tetrahydrobenzofuran-2(4H)-one | 12.78 | C_11_H_16_O_2_ | 181.1215 [M + H]^+^ |  |

Table S3. Molecular docking scores of identified compounds against SARS-CoV-2 Mpro.

| Entry | Compounds | Docking score | PDB ID |
| --- | --- | --- | --- |
| 1 | p-Anisaldehyde | -4.0 |  |
| 2 | Nootkatone | -5.7 |  |
| 3 | Caproic acid | -3.9 |  |
| 4 | Isocantleyine | -5.2 |  |
| 5 | 7-Methoxycoumarin | -5.1 |  |
| 6 | 5,3'-Dihydroxy-6,7,4'-trimethoxy flavanone | -7.1 |  |
| 7 | (*R*)-3-phenoxy-1,2-propanediol | -4.9 |  |
| 8 | Isoferulic acid | -5.4 |  |
| 9 | Eupatorin | -7.0 |  |
| 10 | Eleutheroside B | -6.7 |  |
| 11 | Macranthoin F | -7.6 |  |
| 12 | Apiin | -7.7 |  |
| 13 | 2,3-Dihydrobenzofuran | -4.1 |  |
| 14 | Umbelliferone | -5.0 |  |
| 15 | Caffeic acid | -5.5 |  |
| 16 | Cynaroside | -8.4 |  |
| 17 | Luteolin-7-O-glucuronide | -7.7 |  |
| 18 | Crenatoside | -7.6 |  |
| 19 | Verbascoside | -8.4 |  |
| 20 | Daucosterol | -6.7 |  |
| 21 | Loliolid | -5.2 |  |
| 22 | 3,4-dicaffeoylquinic acid | -8.1 |  |
| 23 | luteolin | -7.7 |  |
| 24 | Rubinaphthin A | -7.4 |  |
| 25 | 6-Methyl-3,5-heptadien-2-one | -4.1 |  |
| 26 | 4-tert-Butylcatechol | -4.9 |  |
| 27 | Ermanin | -7.1 |  |
| 28 | (S)-4,4,7a-Trimethyl-5,6,7,7a-tetrahydrobenzofuran-2(4H)-one | -4.9 |  |

Table S4. Predicted ADMET properties of the lead compounds.

| Properties | Model name | verbascoside | 3,4-dicaffeoylquinic acid |
| --- | --- | --- | --- |
| Absorption | Caco-2 | 0.002 | 0.007 |
|  | HIA | 0.289 | 0.481 |
|  | Pgp | 0.522 | 0.537 |
| Distribution | BBB | 0.09 | 0.126 |
|  | PPB | 0.641 | 0.839 |
|  | VDss | -0.478 | -0.025 |
| Metabolism | CYP3A4 inhibitor | 0.013 | 0.048 |
|  | CYP2D6 inhibitor | 0.019 | 0.039 |
| Toxicity | hERG | 0.291 | 0.393 |
|  | hepatotoxicity | 0.554 | 0.415 |
|  | acute oral toxicity (LD50) | 1.92 | 2.315 |
